# A methodological approach to compare continuous and instantaneous sampling and two methods to deal with animals out of sight on dairy cattle behavior and interaction with their calf in the first hours post-partum

**DOI:** 10.3389/fvets.2024.1360239

**Published:** 2024-03-14

**Authors:** Claudia Manfrè, Monica Battini, Alberto Simonetto, Barbara Contiero, Lorenzo Serva, Silvana Mattiello, Stavros Ntalampiras, Simona Normando, Marta Brscic

**Affiliations:** ^1^Department of Animal Medicine Production and Health, University of Padova, Legnaro, Italy; ^2^Department of Agricultural and Environmental Sciences, University of Milan, Milan, Italy; ^3^Department of Agronomy, Food, Natural Resources, Animals and Environment, University of Padova, Legnaro, Italy; ^4^Department of Computer Science “Giovanni Degli Antoni", University of Milan, Milan, Italy; ^5^Department of Comparative Biomedicine and Food Science, University of Padova, Legnaro, Italy

**Keywords:** animal welfare, dairy cattle, dam-calf bond, maternal behavior, social cognition, vocalization

## Abstract

Animal behavior can provide useful information about animal welfare, but methods and tools used to gather behavioral data and data treatment can influence the results. Therefore, this study was carried out on dairy cow (*Bos taurus*) behavior and interaction with calves early post-partum aiming at comparing two sampling rules, namely continuous and instantaneous sampling at scan intervals of 30 s, 1, 2, 3, 4, 5, and 10 min, and of two methods to deal with out of sight animals. The study was based on three assumptions: (1) continuous sampling provides the most complete and accurate data, allowing the observation of seldom behaviors and short events; (2) instantaneous sampling can provide accurate measurements of frequency and duration, especially at short scan intervals; (3) differences in behavioral results may occur depending on whether a correction for time out of sight is applied or not. Thus, 10 dams were observed from videos in the 2 h post-parturition. Ruminating, stereotypies, calf-biting and calf-butting were not recorded during the observation period. Other behaviors were observed only with continuous sampling or with continuous and instantaneous at 30-s scan intervals. The recoding of several behaviors was less accurate when applying longer scan intervals. Data from continuous and instantaneous sampling at 30-s scan intervals were compared with Wilcoxon test. Results showed no significant differences for posture, position in the pen and all behaviors (*p* > 0.05) except vocalizing (*p* = 0.003). The same test did not highlight significant differences due to method of dealing with out of sight for both sampling rules (*p* > 0.05). Correlation between continuous and instantaneous sampling were prevalently high at 30-s intervals and they decreased as the length of scan intervals increased for most behaviors. Results confirmed the first two assumptions suggesting that continuous sampling is more accurate, in particular for short and rare behaviors, and caution against the suitability of dam behavioral data collected using instantaneous sampling at scan intervals of minutes. The third assumption was not proven by this study. Results should be considered in light of the development of new technologies that relies on data acquired by sensors and imaging to monitor cow-calf welfare and behavior post-parturition.

## 1 Introduction

Animal behavior has received particular attention since ancient times and human evolution took advantage of domestication making the animal-human connection so deep to consider it unbreakable (1). Shipman (1) claims that toolmaking, language and symbolic behavior, and domestication of other species are the three big advances that humans achieved for their evolution. This made them develop abilities such as detection of animals’ behavior and dynamic styles of interaction with individuals of their own and other species, and surrounding environment. Different ways to assess animal behavior are used in ethology, based on the fact that the methods by which data are gathered can influence the results (2, 3). Some studies compared methods in applied ethology, in primatology in particular (4–6). In farm animals, Chen et al. (7) analyzed in detail behaviors of free stall housed dairy cattle related to the physical/functional domain. Madruga et al. (8) compared different recording rules when studying the behavior of growing heifers housed individually and fed a high-concentrate diet. Other methodological behavioral studies were conducted on farmed foxes (9). To the best of authors’ knowledge, no study investigated dams’ behavior after parturition applying different behavior sampling rules and different methods to deal with out of sight.

According to Lehner (10), the most accurate behavior sampling rule is continuous sampling (CS) that is also considered the gold standard. Continuous sampling consists of observing and recording every animal behavior for the whole duration of the selected period, leading to a complete and accurate estimation of both frequencies and durations. It also allows the recording of seldom and fast expressed behaviors (e.g., vocalizations). Since CS application is very time consuming to study animal behavior, an alternative method can be applied, namely instantaneous sampling (IS). When applying IS, data are collected at fixed time intervals within a selected time period (2, 10). When time intervals are short enough, especially in relation to the duration of behaviors, IS can provide a reasonably accurate measurement in terms of both frequencies and durations (10, 11). According to Pullin et al. (12), validation is necessary to gather accurate data when applying IS, however. The need to have reliable and valid behavioral data gathering methods can be even more pronounced when studying complex situations with important economic and animal welfare correlates. One example is the early cow-calf separation where the restricted expression of situation-related behaviors (e.g., calf grooming and licking) affects the animal welfare state, as debated by Mellor (13). The suppression of conscious behavioral choices as distinctive characteristics of animals’ agency deprives animals of the positive feelings that accompany agency (14, 15). The domains of affective experience (13) are gaining interest by scientists and lay citizens. Public concern for early separation of the calf from the dam has increased in recent years as well as research on cow-calf contact systems as valid alternatives (16–18). Several studies investigating different aspects of cow-calf contact systems proved beneficial effects of the expression of their natural bonding for both dam and calf (17, 19, 20). Additionally, perspective papers including socio-economic aspects were published to bridge consumer’s demands (21, 22). However, nowadays there is still not enough knowledge to help farmers to implement cow-calf contact systems on a large scale (22). Thus, new-born calves are still conventionally separated from their mothers soon after birth, although recent recommendations suggest to keep the calf with the dam for at least 1 day (23). In this context, there are several reasons why it is necessary to identify adequate behavior sampling techniques to closely investigate dam behavior post-parturition. For example, calf rearing from birth on in cow-calf systems is shifting from controlling the animals (e.g., bottle feeding of calf) to monitoring undisturbed behaviors in the calf care phase in which cows are left alone to care for their offspring (24). The dam invests more in calf bonding in case of parturition when no stockmen are around and has the possibility of being left alone in a quiet environment (23, 25). Hence, monitoring systems for the automatic detection of the welfare of animals, including video imaging, that replace human surveillance (26) may contribute to lower unnecessary stockmen interactions with the bonding pair. In particular when stockmen’ decisions rely on technology, the latter should be validated based on appropriate sampling methods of behavioral observations.

The study is grounded on the following assumptions: (1) continuous sampling provides the most complete and accurate data and it also allows the observation of seldom expressed behaviors and events with short duration; (2) instantaneous sampling can provide accurate measurement in terms of both frequency and duration, especially at short scan intervals, with shorter intervals providing the most accurate results, comparable with continuous sampling (10); (3) differences in behavioral results may occur depending on whether a correction for time out of sight during video-recordings is applied or not. These potential differences may have implications for automated systems due to the need for correcting data (visible *vs* not visible subject). For several behaviors direct observations are more reliable than video recordings, although animal out of sight occurs also during direct observations.

On the basis of these assumptions, the present paper aims to compare two different sampling rules, namely continuous and instantaneous sampling at different scan intervals (30 s, 1, 2, 3, 4, 5, and 10 min) and to compare two methods of data treatment (inclusion *vs* exclusion of out of sight animals) on the behavior of dairy cows and interaction with their calf immediately after parturition.

## 2 Materials and equipment

The present methodological study is part of a wider research action on cow-calf management. As a first step, this project responded to the need to assess the effects (if any) that data acquisition methods could have on the results of future studies targeting cow behavior and interaction with the calf in the first hours after parturition. To this end we run the methodological study outlined in this paper.

### 2.1 Study area

The study was conducted at the teaching farm “L. Toniolo” of the University of Padua, Legnaro (PD) Italy (45.35209546148253, 11.951079953766982). The farm hosted 36 lactating cows of the Pezzata Rossa Italiana (PRI) breed and about the same number of replacement stock.

### 2.2 Animal housing

Lactating cows are housed in a free stall barn with 62 straw bedded cubicles (proportion with number of cows = 1.7) with access to an outdoor loafing area. Pregnant heifers and dried off cows are housed in a separate group in the same cubicle area and have access to a rotational pasture area. Periparturient animals are housed in a dedicated fully littered parturition pen of 40 m^2^ that may host one or two cows. Replacement heifers are housed indoors in four group pens homogeneous for weight and age. Calves, separated from their dams, are housed in straw-littered individual pens (2.10 m width × 1.60 m length) at ground level in the same replacement stock indoor barn. In accordance with the European Council Directive 2008/119/EC, contact among calves in contiguous pens is allowed through the bars and at the manger that is a continuous space in front of the calves.

### 2.3 Life routines

The farm is run by a farm manager (AS), three permanent farm personnel and two seasonal employees. Lactating cows are milked twice a day in a fishbone milking parlor and have an average milk yield of 29 liters/day/head. Female calves of PRI breed are usually kept as replacement stock, whereas male calves or crossbred females are sold at an age over 1 month at a local farm for beef production. Pregnant cows and heifers are usually dried off 60 days before the expected parturition day and 4 days before they are moved to the parturition pen. In case more cows are expected to deliver in the same period, a contiguous empty pen is also littered and used as a parturition pen. Usually, calves are separated from their dams from 15 min after calving to several hours, depending on the moment when the calf is born and noticed by the farm personnel. After separation, calves do not have access to their dams anymore and no olfactory nor visual contact between dam and calf is possible. Depending on the moment of separation, calves are managed by the farm personnel. The first colostrum meal is fed at calf separation and consists of 3 liters provided via nipple bottle, either milked from the dam or thawed from the colostrum bank. A gastric tube is used for colostrum feeding only if the calf refuses the meal or has difficulties in ingesting it.

This study did not interfere in any way with the common husbandry practices normally carried out on the farm, thus, no changes in the management were required. The cows were moved in and out of the dedicated parturition pen and calves were managed and separated from their dams at times established by the farm personnel, and all practices recorded were in line with current legislation on the protection of farmed animals (27).

### 2.4 Study animals

Ten periparturient animals of the PRI breed were involved preliminarily in the current methodological study. They were selected as a convenience sample, delivering over 6 months from December 2020 to June 2021.

Data regarding the animals and their calving were collected through a questionnaire filled in by the farm manager. It regarded information about the dam (e.g., ID code, parity, possible difficulty at calving), the calf (e.g., ID code, sex, possible gastrointestinal or respiratory or other pathologies) and the colostrum management. Some detailed information about the 10 dams is reported in Table 1. Five dams delivered during the day between sunrise and sunset, and five delivered during the night between sunset and sunrise. After parturition, the 10 dams spent with their calves a period ranging from 20 min to 7 h and 25 min, according to management practices and the moment of separation defined by the farm personnel. All dams were pluriparous except for one primiparous. In two cases the intervention of stock people was necessary, whereas all other deliveries were spontaneous with no human intervention. The 10 dams gave birth to calves of both sexes, and one of them gave birth to male twins. No cases of stillbirth nor maternal rejection of the calf were recorded in the timeframe of the current study.

**Table 1:** Information about the 10 dams included in the study.

Dam information		
**Parturition time**		
	Day	D3, D4, D7, D8, D9
	Night	D1, D2, D5, D6, D10
**Parity**		
	1apex	D10
	2apex	D7, D8, D9
	3apex	D5
	4apex	D6
	5apex	D3, D4
	7apex	D1, D2
**Intervention of stock people**		
	Necessary	D3, D7
	Not necessary	D1, D2, D4, D5, D6, D8, D9, D10
**Calves sex**		
	Male	D3, D6, D7
	Female	D1, D2, D4, D5, D8, D9, D10
**Twin birth**		
	Yes	D3
	No	D1, D2, D4, D5, D6, D7, D8, D9, D10
**Time with calf**		
< 1 h		D4, D7, D8
> 1 h		D1, D2, D3, D5, D6, D9, D10

### 2.5 Video recordings

For the purpose of this study, dam behavior was recorded using a digital HCVR color video camera positioned under the ceiling in a corner above the parturition pen. Most of the pen was visible during the observation time with the only exception of an area under the video camera that was out of sight, where a water trough drinker with a floating valve was positioned. Each cow was video recorded for a total of 5 h, starting 1 h before calving and ending 4 h after. Video recordings were stored as digital files and reproduced at normal speed using Smart Player (media player for Windows version 10, Microsoft Corporation, Redmond, WA) for data extraction without using any specific behavior observation software.

## 3 Methods

### 3.1 Working ethogram

The first step was to define a working ethogram. A first draft was based on scientific literature (8, 28–31). Additionally, as reported in Table 2, behaviors were defined using descriptive terms that allow their identification and categorization. The working ethogram used was functional for the description of dam maternal behavior, interactions with the calf and other behaviors. It included also non-mutually exclusive behaviors that may have occurred at the same time (e.g., looking out of the pen and sniffing air; locomotion and self-grooming; nursing and placenta ingestion). In this case only one of the non-mutually exclusive behaviors was recorded. Given the small sample size and the limited period of observation, some maternal behaviors, interactions with the calf and other behaviors around parturition could be not expressed by the animals, and therefore they were not recorded and analyzed in this study.

**Table 2:** Working ethogram used for the observations of 10 dams in the first 2 h post-parturition.

Category/Behavior	Code	Definition	Reference
**Affiliative behaviors toward the calf**			
Sniffing	Sniff_Calf	Dam has the muzzle in close proximity (< 5 cm) of the calf and inhales and exhales air through the nose in short repetitive manner near the calf in any body part	
Grooming	Gr_Calf	Dam has the muzzle in close proximity of the calf and licks (e.g., puts her tongue repetitively in contact with) the calf in any body part	
Nudging	Nudge_Calf	Dam pushes, shakes or rubs the calf by touching gently any body part with muzzle or head in an apparent effort to stimulate the calf to stand up	
Nursing	Nurse_Calf	Dam allows the calf to go with the muzzle under her belly and to suckle from the udder	
**Agonistic behaviors toward the calf**			
Biting	Bite_Calf	Dam opens and quickly closes the jaws with the teeth grasping the skin of the calf in any body part	
Moving away or displacing	Displ_Calf	Dam goes away from the calf or pushes the calf away by a forceful contact that results in the calf moving away	
Butting	Butt_Calf	Dam hits the calf with the head (forehead or nose)	(28)
**Calf searching**			
Sniffing the litter	Sniff_Litter	Dam has the muzzle in close proximity of litter and inhales and exhales air through the nose in short repetitive manner near it	
Looking out of the pen	Look-Out	Dam goes to the fence, leans out and looks out of the pen	
Restlessness	Restless	Dam moves restlessly in the pen, walks back and forth, does not lie down	
**Environmental interaction**			
Vigilance	Vigil	Dam is alert, keeps her eyes open and looks around, head position at an angle above the horizontal to the withers, ears upright	(30)
Exploration and interaction with pen fixtures	Expl	Dam sniffs and/or licks on floor, wall or fence and interacts with pen fixtures (e.g., including rubbing herself)	Adapted from (28)
**Situation related activities**			
Placenta ingestion	Placenta	Dam puts in the mouth and chews placenta and birth fluids	
Vocalization	Vocalize	Dam emits vocalizations, audible moos with open mouth and quiet humming with closed mouth, with no distinction between high- or low-pitched vocalizations	Adapted from (29)
Locomotion	Locom	Dam performs movement of legs	Adapted from (29)
Resting	Rest	Dam lays down or sleeps with eyes shut while recumbent	
Inactive	Inact	Dam is awake and, regardless of posture, she is not committed in any activity	
Self-grooming	Self-Gr	Dam performs body care by licking her body and wiping her nose and stretches the own body	Adapted from (30)
Stereotypies	Stereot	Dam is engaged in stereotyped behaviors (e.g., tongue playing, tongue rolling and other non-nutritive oral behaviors), repeatedly nibbles/chews at the fixtures of the pen in a stereotyped way	
**Feeding**			
Eating	Eat	Dam brings food to the mouth and chews and swallows it in the manger	
Rumination	Ruminate	Dam is committed in the regurgitation, mastication, and swallowing of the bolus	(8)
**Other**			
Not visible	Not-Vis	Dam is out of sight being in a position that is either not visible from the camera frame, or the activity is not observable (view blocked, no contrast or for other reasons)	
Other	Other	Dam is committed in behaviors other than those described which are not relevant for the purpose of the present study	
*Modifiers adding information about a behavior*			
**Posture (complementary)**			
Standing	Stand	Dam remains on the four limbs without other parts of the body in contact with the ground	Adapted from (31)
Lying	Lying	Dam stays on the litter, on sternal or lateral recumbence, head can or cannot be in contact with the ground, eyes can be open or closed	Adapted from (31)
**Position in the pen (complementary)**			
Front	Front	75% of the body of dam is located in the front half of the pen	
Back	Back	75% of the body of dam is located in the back half of the pen	
**Locked in the feeding rack**			
Locked in the feeding rack	Locked	Dam is blocked with the head in the manger with the use of the feeding rack lock or tied for a short time	
**Contact with the calf**			
Visual contact	Vis-Contact	Dam maintains eye contact with the calf before separation, when physical contact is broken (she looks at the calf, but does not maintain physical contact because she is committed in other activities) or during separation (she follows the movements of the calf with her eyes and/or with the body)	
Physical contact	Phys-Contact	Dam maintains physical contact with the calf by standing or lying down in contact with the calf	
No contact	No-Contact	Dam does not maintain contact, neither physical nor eye contact with the calf	

### 3.2 Preliminary intra-and inter-observer reliability

In order to apply the ethogram in the same way, intra- (CM) and inter-observer (CM, MB) reliability tests were run, preliminarily to the data extraction for this study. Reliability was tested using two random samples of 30-min video clips of two cows (one per each cow) that were not used for the data extraction included in this study. Intra-observer reliability within the same observer who extracted the data over time was tested during July 2021 and February 2022 with a randomized order of application of the two continuous and instantaneous sampling rules. Inter-observer reliability test was performed by the trained assessor in charge of the behavioral data extraction (CM, master level animal science student) and the trainee (MB, veterinarian with experience in behavioral observations, Dipl. ECAWBM-AWSEL) independently. The two observers remained blinded regarding the data extracted by each other by sending the data directly to the co-author in charge of statistical analysis (BC).

### 3.3 Behavioral data

The behavioral data extraction was carried out by one trained observer (CM) using the working ethogram reported in Table 2. The observations regarded exclusively the dam that was the focal animal. For this study, a time frame of 2 h, starting from the moment of expulsion of the calf, was selected regardless of the fact that the dam and her calf had been separated or were still together. Behavioral data were extracted filling in two different recording sheets applying the two different sampling rules (one per each sampling rule).

One rule was continuous sampling (CS), in which each dam was observed continuously for 2 h and all behaviors were recorded with relative start and end times along with the associated modifier (if applicable). Potential modifiers were postures, position occupied in the pen, contact with the calf and time spent locked in the feeding rack. For some behaviors it was not possible to be associated to a modifier (e.g., eating in the back of the pen was not possible since the manger was in front of the pen). Start and end times of a given behavior were recorded as two bouts at the change of a modifier (e.g., placenta ingestion with no contact with the calf and placenta ingestion with visual contact with the calf).

The second rule was instantaneous sampling (IS), in which the same behaviors, postures, and positions in the pen were recorded at 30-s scan intervals. Both observation methods were applied on the same videos for each dam and the total 2-h observation time was split into four 30-min time periods (A from 0 to 30 min; B from 30 min:1 s to 60 min; C from 60 min:1 s to 90 min; D from 90 min:1 s to 120 min) in order to compare the two sampling rules within similar conditions (cow with or without the calf) to limit the large variation of the time spent with the calf in the farm involved in the study. The order of observation of the videos (1 to 10) and the method applied (CS and IS) were established following a 10 × 2 entry table assigning a random order obtained using a random number generator.

### 3.4 Data treatment

Behavioral data recorded using the CS method were transformed into percentage of time spent in each behavior during the four 30-min time periods (A, B, C, D). Moreover, CS data were used to calculate mean bout duration of each behavior and bout frequency. Behavioral data collected through IS every 30 s were transformed from absolute scan frequencies for each behavior into percentages of scans in which the animal was observed engaged in that specific behavior on the total number of scans for each time period (A, B, C, D). The datasets of behaviors recorded at scan intervals of 1, 2, 3, 4, 5 and 10 min were obtained from data collected through IS at 30-s scan intervals.

### 3.5 Statistical analysis

All data were insert in Excel spreadsheets (Microsoft Office, Microsoft Corporation, Redmond, WA) and all statistical analysis were performed using SAS (SAS Inst. Inc., Cary, NC, United States).

#### 3.5.1 Intra-and inter-observer reliability tests

Intra-and inter-observer reliability tests were done on behavioral data gathered with both continuous and instantaneous (30-s intervals) sampling rules using Kendall Correlation Coefficient W. Kendall W values of 0 mean no agreement, values higher than 0.6 mean substantial agreement and values of 1 mean complete agreement. The behaviors failing the intra- or the inter-observer reliability test (W < 0.60, *p* > 0.05) were excluded from the study (see results section).

#### 3.5.2 Comparisons between rules and methods

Behaviors that were not observed in the first 2 h after parturition were excluded from analysis, along with the complementary posture and position in the pen for which only one of the two was analyzed (standing and front).

Data were at first submitted to descriptive statistical analysis and when applicable (observed with both sampling rules/methods), two comparisons were carried out:

1. Between percentages of time/scan spent in each behavior, posture and position in the pen in the four 30-min intervals obtained using two different sampling rules (i.e., CS *vs* IS at different sampling intervals) and the same method of dealing with animals out of sight;
2. Between percentages of time/scan spent in each behavior, posture and position in the pen in the four 30-min intervals obtained using two different methods of dealing with animals out of sight (total *vs* visible) and the same sampling rule.

##### 3.5.2.1 Comparison between sampling rules

In order to compare outcomes of the two sampling rules (CS and IS), behavioral data from CS (expressed as percentage of time in which the animal was observed in each complementary posture and position in the pen, and engaged in each behavior) were compared with data from IS (expressed as percentage of scans with scan intervals of 30 s) during the four 30-min time periods, applying the Wilcoxon pairwise test. The Wilcoxon test is a non-parametric statistical test that compares two paired groups aiming at determining whether two or more sets of pairs are different from each other in a statistically significant way.

Behavioral data from CS were correlated with data from IS expressed as percentage of scans with scan intervals of 30 s, 1, 2, 3, 4, 5 and 10 min using the Spearman rank correlation. Correlations between the two sampling rules were classified according to the r value (high: 0.75 to 1 or − 0.75 to −1; moderate: 0.5 to 0.75 or − 0.5 to −0.75; weak: 0.25 to 0.5 or − 0.25 to −0.5; very weak: 0 to 0.25 or 0 to −0.25) considering the values proposed by Munita et al. (31). Additionally, to evaluate the accuracy and bias of each sampling interval, a linear regression analysis was conducted. For each posture, position in the pen and behavior, pairwise comparisons were made between the behavioral estimates from each sampling interval (30 s and 1, 2, 3, 4, 5, and 10 min) and the CS data. A tested sampling interval was considered to accurately estimate the behavior if the following criteria were met: *R*^2^ ≥ 0.90, slope not statistically different from 1 (*p* > 0.05), and intercept not statistically different from 0 (*p* > 0.05), as suggested by Pullin et al. (12). The combination of these values reflects the strength of association (R^2^), linear relationship (slope), and over- or underestimation of the duration values of each behavior (intercept) (32, 33).

##### 3.5.2.2 Comparison between total time *vs* visible time

Due to the methodological nature of the present study, a comparison was done also between two methods of dealing with subjects out of sight (34) on data gathered with both CS and IS at 30-s scan intervals. To this end, percentage durations calculated on total time (as explained above; total percentage) were compared to those calculated only on the time that the animal was visible (observed and not out of sight; visible percentage), as suggested by Lehner (34) for subjects disappearing from view. Spearman rank correlations and the Wilcoxon pairwise tests were run to assess, for each behavior, associations and differences between total and visible percentages resulting from the same sampling rule. Additionally, to evaluate the accuracy and bias of each method, a linear regression analysis was conducted.

## 4 Results

### 4.1 Intra-and inter-observer reliability test

Intra-observer reliability on CS data resulted in a complete agreement (*W* = 1, *p* < 0.001) for standing, calf grooming, calf nudging, eating, and resting in the 30-min videos used for the test. All other behaviors showed a substantial agreement (*W* ≥ 0.63, *p* ≤ 0.028) with the exception of calf sniffing (*W* = 0.57, *p* = 0.052). Inter-observer reliability test on CS data revealed complete agreement (*W* = 1, *p* < 0.001) for standing, whereas a substantial agreement (W ≥ 0.69, *p* ≤ 0.012) was found between observers for most other behaviors, except for vigil (*W* ≤ 0.29, *p* ≥ 0.368), inactive (*W* = −0.36, *p* = 0.251), and other behaviors not included in the ethogram (*W* = 0.27, *p* = 0.405).

The intra-observer reliability test gave a complete agreement also for self-grooming and not visible when sampled using IS. It was not applicable to calf nudging and calf sniffing that were not observed using IS in the 30-min videos used for the test. All other behaviors showed a substantial agreement (W ≥ 0.63, *p* ≤ 0.028). In comparison with CS results, the inter-observer reliability test revealed a complete agreement for calf grooming and resting. Most other behaviors showed a substantial agreement (*W* ≥ 0.69, *p* ≤ 0.012), except for vigil (*W* ≤ 0.29, *p* ≥ 0.368) and locomotion (*W* = 0.03, *p* = 0.917). The test resulted not applicable for inactive and other behaviors.

### 4.2 Descriptive statistics of behavioral data gathered with continuous sampling

Rumination, stereotypies, biting and butting the calf were not observed in the first 2 hrs after parturition in any of the videos object of the current study. Drinking was excluded for methodological reasons due to the fact that the drinker was out of sight.

**Figure 1:**
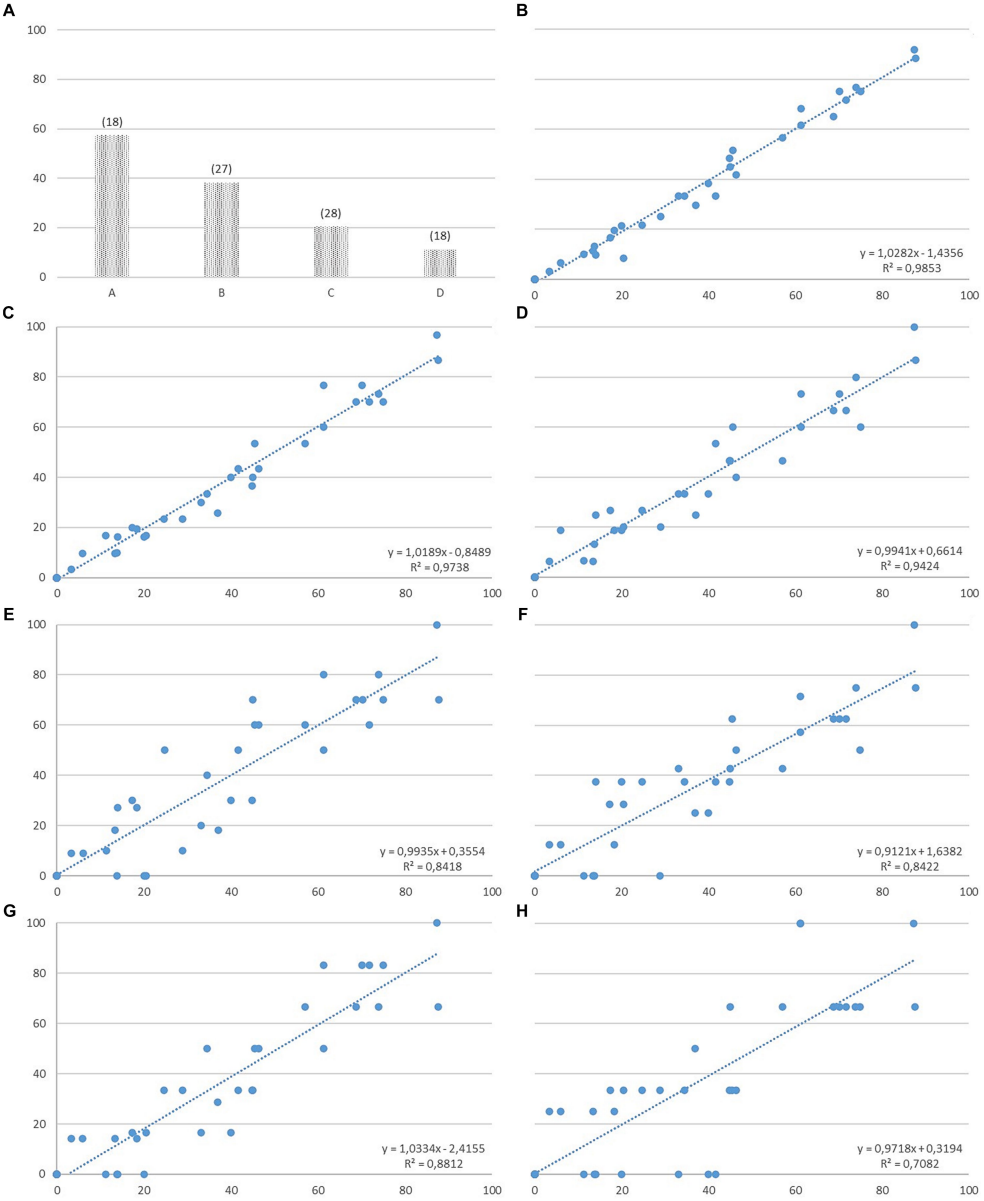
Mean percentage of time spent (%) and number of bouts (in brackets) of calf grooming extrapolated using continuous sampling of 10 dams during four 30-min periods (A, B, C, D) covering the first 2 hours after parturition on total data **(A)**; and graphical representation of the regression analysis for calf grooming between data obtained using CS (% of time) and IS at 30 s **(B)**, 1 min **(C)**, 2 min **(D)**, 3 min **(E)**, 4 min **(F)**, 5 min **(G)**, and 10 min **(H)** scan intervals (% of scans) on total data (not correcting for out of sight).

The dams spent most of the two-hour observation time in standing posture with a mean percentage of 84.4% of the total time. The position chosen by the dams in the parturition pen was prevalently the back where they spent 54.0% of the time. Four cows were, however, locked at the feeding rack as a commonly adopted managerial practice spending 12.7% of the total time there. Dams kept visual and physical contact with their calves in particular during the first 30-min interval after parturition and contact gradually decreased over the course of the 2 h. During intervals C and D, there were no contacts between cows and calves separated within the first hour. Regardless of the time spent with the calf, over the 2 h after parturition, dams had visual and physical contact with their calf for a mean of 33.9 and 37.2%, respectively. These percentages of contact with the calf were 49.5 and 53.7% for visual and physical contact respectively, if calculated in relative terms within the timeframe when the dam had the calf present in the parturition pen. Among maternal behaviors, calf grooming was the most prevalent. All dams were observed engaged in calf grooming right after parturition, and they spent a mean of 31.5% of time in this behavior during the two-hour observation time (Figure 1A). Calf grooming was interrupted when separation took place, while the dams that were still with their calves continued to exhibit it with a progressive reduction over the observation time. Three dams (D5, D9 and D10) were engaged in calf nudging for 1.9% of the time with a mean bout duration of 8.1 s. Only one dam (D1) was observed nursing her calf in a single bout lasting 25 s during time interval D. In the 2 h after parturition, six dams were observed ingesting the placenta and birth fluids at different time intervals (D1, D2, D3, D4, D7, and D10) whereas the remaining were either not observed engaged in placetophagia or they did not expel it by the time of observation. All dams were observed emitting vocalizations with a mean bout duration of 1.8 s. The number of bouts in which dams vocalized was greater when dams were together with their calves. Some behaviors occurred with a very low overall percentage, and they were: displacement of the calf (0.08%) and interaction with the environment (0.3%). Dams separated from their calves were observed more often engaged in sniffing the litter. They were the only dams looking out of the parturition pen and eating, and they spent more time resting, in particular during time interval D. Two dams exhibited visible signs of restlessness in the first hour post parturition: D4 during interval A and D7 during interval B. Vigilance behavior was manifested by all dams, with increasing trend over intervals A, B and C, and the number of bouts recorded was greater for dams with calves. Time out of sight was an overall mean of 16.9% with a mean bout duration of 29.5 s.

### 4.3 Comparison between rules and methods

#### 4.3.1 Comparison between sampling rules

Correlation testing were performed on total data (without correcting for out of sight time). Restlessness was observed only when applying CS and was not recorded when applying IS, thus it was not analyzed in the comparison between sampling rules. Nursing was observed only once when applying CS and IS, but only at 30-s scan intervals; longer scan intervals did not allow the recording of this behavior.

Spearman’s rank correlations analysis comparing percentages of posture, position in the pen and behaviors recorded using CS and IS at different scan intervals (30 s, 1, 2, 3, 4, 5, and 10 min) are reported in Table 3. Most behaviors showed high correlation values between CS and IS at 30-s intervals. Standing, spending time in front of the pen and calf grooming highly correlated between the sampling rules, regardless of the duration of the scan interval. For most behaviors (e.g., eating, placenta ingestion, self-grooming, resting, sniffing the litter and not visible) the correlation values decreased as the time interval increased. Other behaviors were not comparable between sampling rules when the scan interval lasted minutes. Results of the linear regression analysis, conducted to evaluate the accuracy and bias of each sampling interval, revealed that the 30-s sampling interval accurately estimated posture, position in the pen and all behaviors (*R*^2^ ≥ 0.90, *p* < 0.001) except for calf displacement (*R*^2^ = 0.61, *p* < 0.001), vocalization (*R*^2^ = 0.28, *p* = 0.001) and exploring (R^2^ = 0.88, p < 0.001). The accuracy of the estimate was not reached for IS data at scan intervals of 10 min for neither posture (*R*^2^ = 0.73, *p* < 0.001), position in the pen (*R*^2^ = 0.89, p < 0.001), nor for any of the behaviors (*R*^2^ ≤ 0.89, *p* ≤ 0.023). The combination of the *R*^2^, slope and intercept values reflecting the strength of association, linear relationship, and over- or underestimation of the engagement in each behavior showed that the slope began to vary from 1 at IS intervals of 1 min or longer. As an example, the graphical representation of the regression analysis for calf grooming is reported in Figure 1, along with its descriptive statistics related to percentage of time and number of bouts recorded using CS.

**Table 3:** Results (ρ) of the Spearman’s rank correlation analysis comparing posture (Standing), position in the pen (Front) and behaviors recorded with continuous sampling and instantaneous sampling at different intervals [30 s, 1, 2, 3, 4, 5, and 10 min] on total time (not correcting for out of sight).

Continuous sampling		Standing	Front	Grooming	Nudging	Nursing	Moving away or displacing	Eating	Placenta ingestion	Self-grooming	Resting	Vocalization	Exploration and interaction with pen fixtures	Sniffing thelitter	Looking out of the pen	Not visible
Instantaneous sampling	30 s	0.97***	0.99***	0.99***	0.74***	1.00***	0.72***	0.96***	0.84***	0.86***	0.99***	0.70***	0.79***	0.92***	0.77 **	0.97***
	1 min	0.97***	0.99***	0.99***	0.54***	–	0.72***	0.89***	0.84***	0.72***	0.99***	0.39*	0.61***	0.86***	0.66***	0.93***
	2 min	0.94***	0.99***	0.98***	0.54***	–	–	0.83***	0.71***	0.57***	0.84***	0.39*	0.44**	0.80***	0.66***	0.88***
	3 min	0.91***	0.99***	0.92***	–	–	–	0.76***	0.77***	0.43**	0.69***	0.18	0.53**	0.64***	0.66***	0.90***
	4 min	0.91***	0.98***	0.92***	0.37*	–	–	0.68***	0.71***	0.50***	0.69***	0.18	0.44**	0.70***	0.66***	0.84***
	5 min	0.90***	0.98***	0.94***	0.54***	–	–	0.62***	0.51***	0.47**	0.77***	–	–	0.68***	–	0.81***
	10 min	0.80***	0.95***	0.84***	0.39*	–	–	0.62***	0.40**	0.27	0.41**	–	–	0.55***	–	0.72***

*p < 0.05; **p < 0.01; ***p < 0.001; − not applicable.

When applying the Wilcoxon test on the pairwise comparison between CS and IS at 30-s scan intervals, results showed no significant differences for posture (*p* = 0.756), position in the pen (*p* = 0.946) and all behaviors (*p* ≥ 0.216) with the sole exception of vocalizing that showed a significant difference between CS and IS (*p* = 0.003), with CS revealing more vocalizations compared to IS at 30-s scan intervals.

#### 4.3.2 Comparisons between methods used to deal with animals out of sight (i.e., total time *vs* visible time)

**Figure 2:**
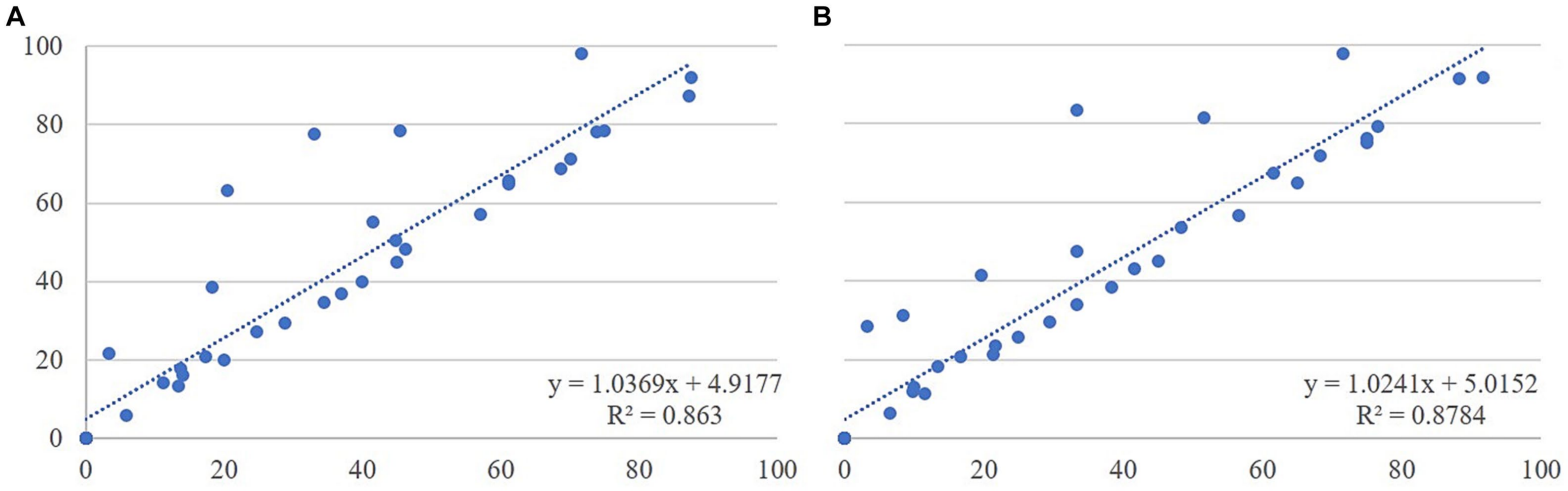
Graphical representation of the regression analysis for calf grooming between total and visible data corrected for out of sight obtained using continuous sampling **(A)** and between total and visible data corrected for out of sight obtained using instantaneous sampling at 30 s scan intervals **(B)**.

The correlations between the data gathered on total and visible time were high, with values ranging from 1 (standing, in front of the pen, resting, looking out of the pen, nursing, nudging, displacing) to 0.95 (calf grooming) for CS and from 1 (standing, in front of the pen, nursing, looking out of the pen, displacing) to 0.95 (calf grooming) for IS at 30 s scan intervals. The linear regression for calf grooming when visible and out of sight is graphically represented in Figure 2. Results of the linear regression analysis between total time and visible time revealed that both methods of dealing with animals out of sight accurately estimated posture, position in the pen and all behaviors (*R*^2^ ≥ 0.90, *p* < 0.001) except for calf grooming (*R*^2^ = 0.86, *p* < 0.001) and vocalization (*R*^2^ = 0.74, *p* < 0.001) when recorded using CS, and calf grooming (*R*^2^ = 0.88, *p* < 0.001), nudging (*R*^2^ = 0.88, *p* < 0.001) and exploration (*R*^2^ = 0.77, *p* < 0.001) when recorded using IS. The combination of the *R*^2^, slope and intercept values showed that there was a robust association, a linear relationship, and no over- or underestimation for standing, in front of the pen, resting, calf displacement and looking out of the pen when comparing the way to deal with out of sight animals within the same sampling method. All the behaviors showed slope values close to 1 (mean ± SD: 1.07 ± 0.09 for CS and 1.12 ± 0.17 for IS at 30 s interval) and intercept values close to 0 (mean ± SD: 0.05 ± 0.12 for CS and 0.03 ± 0.09 for IS at 30 s interval) for both sampling methods, except for calf nursing and calf grooming. The slope value for calf nursing did not show a linear relationship when correcting for out of sight with both sampling methods. Calf grooming had an intercept value above 1 with both sampling methods (Figure 2).

Wilcoxon pairwise test did not highlight any significant difference between analysis on data obtained from the two methods of dealing with animals out of sight for both CS and IS (all *p* > 0.05).

## 5 Discussion

As a preliminary step of an ongoing project on cow-calf management, this methodological study was carried out to assess the effects that data sampling rules (CS *vs* IS at different scan intervals) and methods of dealing with out of sight animals (total *vs* visible time) might have on data on cow behavior and cow-calf interactions in the first hours post-parturition. Potential practical implications of this study merge with the need to identify suitable behavior recording techniques raised by the development of technology-based animal monitoring systems. Automatic detection of animal behavior and welfare and its validation process should rely on appropriate sampling rules and correct methods to deal with animals out of sight. In this context, monitoring systems and technology may be a solution to eliminate observer individual perception which may create bias in the detection of animal welfare and health (35). This is particularly relevant when applied on cow-calf contact systems that are changing from human management of the calves to observations and monitoring of undisturbed dams caring for their calves (24). The management of new-born dairy calves and the provision of different types of cow-calf contact systems is a relevant scientific topic at present (23). Cow maternal behavior and cow-calf interactions have been object of several scientific papers (36–39). Therefore, this study focused on the comparison between the results obtained using different sampling rules and methods of dealing with out of sight to widen the discussion on behavioral observation application to validate precision livestock farming technology and further develop artificial intelligence applied to monitor cow-calf interactions.

As a first step, assessor effect had to be reduced at the minimum achievable level, thus a single observer was engaged and behaviors that showed low agreement at the intra-and inter-observer reliability tests were excluded. These behaviors were those observed with a very low percentage or that occurred in concomitance with other behaviors, because of not being mutually exclusive. It is likely that the observer was paying less attention to them.

Dams spent most of their time standing and in the back of the pen after parturition. Postures and position in the pen turned out to be highly correlated between CS and IS even at scan intervals of 10 min. They showed high correlation values also between methods to deal with out of sight. This is probably due to their relatively long durations, as suggested by Maekawa et al. (40) and Chen et al. (7), but cannot be proven by our results where bout duration of the modifiers was linked to the duration of the specific behaviors they were associated to. The prevailing time spent at the back of the pen is likely mimicking the dam’s instinctive need to search for an isolated undisturbed place to deliver and bond with the calf, away from predators and other members of the herd, as they would do in natural conditions (41, 42). As expected, calf grooming was the prevalent affiliative behavior and it was mainly performed while standing. Calf grooming showed high correlation values between CS and IS at all the considered scan intervals and was not affected by the method to deal with out of sight. Calf grooming was prevalent in the first 30 min post-partum. It was interrupted at separation of the calf from the dams and decreased over the time for dams remaining with their calves. This is likely due to the fact that dams and calves start bonding shortly after parturition (20, 39, 43). Since dams dry and clean their calves by licking them for a prolonged time, lasting several minutes, it could be expected that IS provides a reliable estimate of such behavior even if applying scan intervals longer than 30 s. The greater number of bouts during periods B and C suggests that dams often stopped grooming their calf, likely because they were disturbed by external events. The quality of the bond depends on the possibility of being left alone in a quiet environment (25). Individual parturition pens (44), motivation-based secluded calving areas (45) and bonding pens for the cow-calf pairs in the days following parturition (25) were suggested to ensure a safe calving area and a good-quality bonding.

Less frequent affiliative interactions with the calf, such as nudging, showed a moderate correlation even with IS at 30-s intervals. The correlation values decreased as the length of scan intervals increased. Intervals of 1 min and above could not be considered as reliable for recording calf nudging. In an analogous way, the low reliability revealed between and within observers and with IS for calf sniffing in this study, could also be related to the relatively short duration of this affiliative behavior. This is supported by Lehner (10), who reports that, when sampling intervals are short in relation to the mean duration of the behaviors and the behaviors occur at high rates, then IS can provide accurate measurement of both frequency and duration. However, only CS is able to provide precise and accurate information on events. A similar conclusion was stated by Chen et al. (7) about visits at the feed bunk and drinking events in dairy cattle that seem being accurately reflected only with CS or with IS at short sampling intervals.

Differently from our expectations, nursing was observed only once in the current study. It was not recorded using IS at scan intervals longer than 30 s and the correction method of dealing with out of sight seems affecting it in a non-linear relationship. Cows in the current study were of a dual-purpose breed with a recent genetic selection for dairy production and most of them were pluriparous. Therefore, this low prevalence of nursing behavior recorded in the first 2 h post-partum could be associated to the latency of starting to nurse of two to 6 h reported for specific dairy breeds and the longer latency of starting to nurse for pluriparous cows (39). Parity could explain also the null or low prevalence of agonistic behaviors toward calves in this study, although not statistically tested because not applicable. Edwards and Broom (46) report that aggressive behaviors toward offspring are much more frequent in first calving cows compared to pluriparous. In this study, calf displacement was observed with a low frequency and the behavior highly correlated between CS and IS at 30-s scan intervals regardless of the way to deal with out of sight. No correlation was observed between CS and IS with scan intervals longer than 1 min. Thus, based on the above discussed results, it is advisable to apply CS to effectively record? both affiliative behaviors and aggressive interactions between dam and calf. Alternatively, affiliative behaviors could be grouped together, as done by Wenker et al. (20) in a study on cow-calf contact systems. However, a precise distinction of each affiliative and aggressive behavior might be relevant for future studies aiming at the differentiation of maternal care quality. Protective maternal care was evaluated in beef cows relying on numerous and detailed variables related to maternal care and nursing behaviors (47). However, only Qualitative Behavior Assessment (QBA) seemed integrating the wide range of aspects of an individual animal emotional expressivity (48).

In the present study, vocalizations correlated moderately between CS and IS at 30-s intervals and weaker correlation values were observed at longer scan intervals, probably due to their short duration. When applying CS, frequent and short-lasting vocalizations were recorded over the whole two-hour observation time. It is likely that dams observed vocalizing could have emitted frequent bonding vocalizations when the calf was still with the dam, as supported by Watts and Stookey (49). Dams in this study could have vocalized to call their calf after separation although the short time reduced the probability of a fully established maternal-filial bond (38). The lack of specific analyses and characterization of the vocalizations, in the current study, did not allow to distinguish high emissions as a sign of separation stress between bonded cow and calf pairs (38, 43) or numerous low pitch bonding vocalizations that cows use for the communication and recognition with their calves at an early stage of calf lives (49). This confirms that for behaviors with a low frequency and short duration, like vocalizations, only CS is a suitable option. Regardless of the sampling method and way to deal with out of sight, behavioral observations seem not being enough to effectively evaluate vocalizations (type and objective of them), and other adequate instruments and more specific tools are required for this purpose (50, 51).

Regardless of the way to deal with out of sight, CS and IS at 30-s scan intervals seemed accurate in extrapolating data regarding sniffing the litter and looking out of the parturition pen. The correlation values between CS and IS dropped to moderate or weak with longer scan intervals. This goes again in favor of the application of CS or short scan sampling intervals when assessing cow behavior after parturition, especially when behaviors with a low frequency and short duration need to be assessed. In the current study, other behaviors that might explain the dynamic style of interaction of the dam with the calf in this early phase, such as restlessness and vigilance, gave less promising results. Restlessness was recorded only when applying CS and vigilance gave very low intra-and inter- observer reliability. A possible explanation could be related to the fact that restlessness and vigilance are non-mutually exclusive and they could be manifested in concurrence with other behaviors such as calf grooming, and they are not free of some interpretation and subjectivity. Moreover, these results support the need to apply qualitative behavior observations along with quantitative ones to widen the aspect of dam behavior around parturition (e.g., nesting) and the quality of maternal interaction with the calf as discussed by Ceballos et al. (48).

Another frequent behavior shown by cows after parturition is eating the placenta and high correlation values were found between CS and IS at scan intervals up to 5 min in our study, with no effects due to the method of dealing with animals out of sight. This result suggest that this behavior can be recorded effectively with both sampling rules and correcting method. However, our results regarding placentophagia might be partially biased, due to the fact that some of the dams might have not expelled the placenta in the two-hour observation time of the current study, considering that secondment might take longer (39). Shortcomings of this study are a small sample size and a relatively short duration of the observation time, with the consequent potential missing of rare behaviors. Considering that cow maternal behavior and interaction with the calf is affected by numerous factors (environment, farm management, breed, etc.), we speculate that the variability among dams in this study extended the potential expression of the behavioral repertoire without affecting the comparisons between sampling rules and methods to deal with animals out of sight. This highlighting that the aim of the current study was not to assess factors affecting cow maternal behavior.

In a rapidly changing scenario, where increasing attention is given to farm animals’ welfare, especially within the sensitive topic of dairy cow-calf separation, the results of this study can enhance the precision of future studies, thus reducing the number of conflicting or dubious results due to sub-optimal methodological choices. Despite the small number of dams involved, our findings on the effects of different sampling rules on the quality of the obtained behavioral data caution against the suitability of dam behavioral data collected using IS at scan intervals of minutes. For occasional and rare behaviors, the more appropriate method turned out to be CS and for most of the recorded behaviors IS at 30-s scan intervals worked equally well. Being out of sight or not hardly affected the behaviors recorded. Nevertheless, this not necessarily means that all behaviors of a working ethogram may be not affected by out of sight time. For example, drinking was omitted from the ethogram in this study because the drinker was out of sight.

Precise CS of all maternal interactions would be needed also for the individual differentiation of the quality of the maternal bond. In regard to the correcting method for out of sight, our results do not favor one or the other method, however, it could be speculated that only fully visible animals with a drastic limitation of time out of sight with the use of high-tech hardware would allow a full comprehensive picture of the behavioral patterns in cow-calf interaction. The abovementioned issues should be considered for the development of new technologies that rely more and more often on data acquired by sensors and imaging science for the monitoring of animal behavior and welfare. This is particularly important if actions from the farmers are needed according to thresholds set using artificial intelligence in the anticipation of poor quality of the maternal behavior in the bonding timeframe.

## Data availability statement

The raw data supporting the conclusions of this article will be made available by the authors, without undue reservation.

## Ethics statement

Ethical approval was not required for the studies involving animals in accordance with the local legislation and institutional requirements because This methodological study was conduct in respect of the rules for ethical conduct in scientific research. The study was based on videos of dairy cows in a registered farm that were recorded above the parturition pen. All the practices recorded were in line with current legislation on the protection of farmed animals [European Council Directive (52) and (27)] and the study did not interfere in any way with the common husbandry practices normally carried out on the farm. No research procedure was applied to the animals involved in the study. Written informed consent was not obtained from the owners for the participation of their animals in this study because Farm manager and most of the authors are affiliated to the University of Padova and the livestock belong to the teaching farm.

## Author contributions

CM: Writing – original draft, Writing – review & editing, Formal analysis, Data curation. MoB: Writing – review & editing. AS: Data curation, Writing – review & editing. BC: Writing – review & editing, Formal analysis. LS: Formal analysis, Writing – review & editing. SM: Writing – review & editing. SNt: Writing – review & editing. SNo: Writing – review & editing, Writing – original draft, Visualization, Methodology, Conceptualization. MaB: Visualization, Supervision, Methodology, Funding acquisition, Conceptualization, Writing – original draft, Writing – review & editing, Formal analysis, Data curation.
